# Metabolic and Immune Vulnerability in Critically Ill Patients with Diabetes Mellitus

**DOI:** 10.3390/medicina62020341

**Published:** 2026-02-07

**Authors:** Mădălina Diana Daina (Fehér), Codrin Dan Nicolae Ilea, Cosmin Mihai Vesa, Alina Cristiana Venter, Adriana Vladu, Timea Claudia Ghitea, László Fehér, Cristian Marius Daina

**Affiliations:** 1 Doctoral School of Biomedical Sciences, Faculty of Medicine and Pharmacy, University of Oradea, 1 December Square, 410081 Oradea, Romania; 2 Department of Psycho-Neurosciences and Recovery, Faculty of Medicine and Pharmacy, University of Oradea, 1 December Square, 410081 Oradea, Romania; 3 Department of Preclinical Disciplines, Faculty of Medicine and Pharmacy, University of Oradea, 1 December Square, 410081 Oradea, Romania; 4 Department of Morphological Disciplines, Faculty of Medicine and Pharmacy, University of Oradea, 410073 Oradea, Romania; aventer@uoradea.ro; 5 Department of Surgical Disciplines, Faculty of Medicine and Pharmacy, University of Oradea, 1 December Square, 410081 Oradea, Romania; 6 Pharmacy Department, Faculty of Medicine and Pharmacy, University of Oradea, 1 December Square, 410081 Oradea, Romania

**Keywords:** diabetes mellitus, intensive care unit, ICU mortality, lactate, immune dysfunction, acute organ failure, risk stratification, critical illness

## Abstract

*Background and Objectives:* Diabetes mellitus is frequently encountered in critically ill patients and is associated with increased short-term mortality. However, the biological and clinical determinants of mortality within the diabetic intensive care unit (ICU) population remain incompletely understood. This study aimed to evaluate laboratory parameters at ICU admission and key early ICU course variables, including acute complications and organ support interventions, associated with short-term ICU mortality in critically ill patients with diabetes mellitus. *Materials and Methods:* We conducted a retrospective observational cohort study including adult patients with diabetes mellitus admitted to a tertiary care ICU between January and December 2024. Demographic data, laboratory parameters at ICU admission, acute complications, and ICU interventions were collected. Patients were stratified according to ICU outcome (survivors vs. non-survivors). Univariate and multivariate logistic regression analyses were performed to identify independent predictors of ICU mortality. Model performance was assessed using the area under the receiver operating characteristic curve (AUC/ROC), Hosmer–Lemeshow test, and Brier score. *Results:* A total of 443 critically ill patients with diabetes mellitus were included, of whom 239 (54.0%) died during ICU hospitalization. Non-survivors exhibited higher admission blood glucose, lactate levels, and serum creatinine, as well as lower lymphocyte counts compared to survivors. Acute complications, including sepsis, acute kidney injury, and acute respiratory failure, were significantly more frequent among non-survivors. In multivariate analysis, admission lactate levels (OR = 1.02 per mg/dL increase), mechanical ventilation (OR = 47.30), and hemodialysis (OR = 3.38) remained independently associated with ICU mortality. The predictive model demonstrated good discrimination (AUC = 0.87) and adequate calibration. *Conclusions:* Critically ill patients with diabetes mellitus who do not survive ICU hospitalization present with early metabolic stress, immune dysregulation, and organ dysfunction. Admission lactate levels and the need for advanced organ support are key predictors of short-term mortality, supporting their role in risk stratification within the diabetic ICU population.

## 1. Introduction

Diabetes mellitus (DM) is one of the most prevalent chronic metabolic disorders worldwide and represents a major contributor to morbidity and mortality across a wide range of clinical settings. The increasing prevalence of diabetes, driven by population aging and the growing burden of cardiometabolic disease, has led to a substantial rise in the number of patients with diabetes admitted to intensive care units (ICUs) [1,2,3].

In critically ill patients, diabetes mellitus is frequently accompanied by metabolic dysregulation, chronic low-grade inflammation, immune dysfunction, and pre-existing organ impairment. These alterations may reduce physiological reserve and limit the ability to mount an effective response to acute stress, thereby increasing vulnerability during critical illness. Consequently, patients with diabetes often experience more severe disease courses and worse short-term outcomes when admitted to the ICU [4,5,6,7].

Previous studies have shown that diabetes mellitus is associated with increased ICU and in-hospital mortality. However, most investigations have focused on comparing outcomes between patients with and without diabetes, providing limited insight into the determinants of mortality within the diabetic population itself. As a result, the biological and clinical factors that distinguish survivors from non-survivors among critically ill patients with diabetes remain incompletely characterized [7,8,9,10,11].

Early identification of high-risk diabetic patients is of particular clinical importance, as timely recognition of metabolic stress, immune impairment, and organ dysfunction may allow for optimized monitoring and targeted supportive strategies. Biological markers obtained at ICU admission, together with the development of acute complications and the need for advanced organ support, may provide valuable prognostic information in this vulnerable population [12,13].

Therefore, the present study aimed to investigate laboratory parameters at ICU admission, acute complications, and intensive care interventions associated with short-term mortality in critically ill patients with diabetes mellitus. By focusing exclusively on diabetic patients, this study seeks to elucidate key predictors of ICU mortality and to support improved risk stratification in this high-risk group.

## 2. Materials and Methods

### 2.1. Study Design and Setting

We conducted a retrospective observational cohort study in the Intensive Care Unit of the Bihor County Emergency Clinical Hospital, a tertiary care center providing medical and surgical intensive care. The study included patients admitted between 1 January and 31 December 2024.

The study was designed to evaluate biological and clinical predictors of short-term mortality specifically in critically ill patients with diabetes mellitus.

### 2.2. Study Population

All adult patients (≥18 years) admitted to the ICU during the study period were screened for eligibility. For the present analysis, only patients with diabetes mellitus were included.

Diabetes mellitus was defined based on documented medical history, ongoing antidiabetic treatment (oral antidiabetic agents and/or insulin), or diagnostic blood glucose–based diagnostic criteria at admission. Patients with gestational diabetes or secondary forms of diabetes mellitus were excluded.

The final study cohort consisted of 443 critically ill adult patients with diabetes mellitus. Patients were stratified according to ICU outcome into survivors and non-survivors, based on mortality during ICU hospitalization.

### 2.3. Data Collection

Data were extracted retrospectively from electronic medical records and ICU charts. The following categories of variables were collected:

#### 2.3.1. Demographic and Clinical Data

- Age
- Sex
- Type of ICU admission (medical or surgical)

#### 2.3.2. Laboratory Parameters at ICU Admission

Only laboratory values obtained at the time of ICU admission were considered, to reflect the initial physiological status. These included:

- Blood glucose (mg/dL)
- Serum lactate (mg/dL; 1 mmol/L ≈ 9 mg/dL)
- Absolute lymphocyte count (10^3^/μL)
- Serum creatinine (mg/dL)

Lactate values were reported in mg/dL according to local laboratory standards; approximate mmol/L equivalents are provided to facilitate clinical interpretation.

#### 2.3.3. Acute Complications During ICU Stay

The occurrence of major acute complications was recorded as binary variables (yes/no):

- Sepsis
- Acute kidney injury
- Acute respiratory failure

#### 2.3.4. Intensive Care Interventions

The need for advanced ICU interventions during hospitalization was documented:

- Mechanical ventilation
- Hemodialysis

### 2.4. Outcome Definition

The primary outcome was mortality during ICU hospitalization, defined as death occurring at any time during the ICU stay.

### 2.5. Definitions of Acute Complications

Sepsis was defined according to Sepsis-3 criteria based on documented infection and organ dysfunction. Acute kidney injury was defined using KDIGO criteria based on serum creatinine changes during ICU stay. Acute respiratory failure was defined as the need for invasive or non-invasive ventilatory support due to hypoxemia or hypercapnia.

### 2.6. Statistical Analysis

Continuous variables were assessed for normality using visual inspection and were reported as mean ± standard deviation. Categorical variables were expressed as absolute numbers and percentages. Comparisons between survivors and non-survivors were performed using Student’s *t*-test for continuous variables and the chi-square test or Fisher’s exact test, as appropriate, for categorical variables. The proportion of missing data per variable was low (<5% for all included laboratory parameters).

To identify predictors of ICU mortality among patients with diabetes mellitus, univariate logistic regression analyses were first performed for each candidate variable. Variables showing statistical significance in univariate analysis or considered clinically relevant were subsequently included in a multivariate logistic regression model.

Missing data were handled using simple imputation, with median imputation for continuous variables and most-frequent value imputation for categorical variables, to preserve the full cohort size.

Results were reported as odds ratios (ORs) with 95% confidence intervals (CIs). Model discrimination was assessed using the area under the receiver operating characteristic curve (AUC/ROC). Model calibration was evaluated with the Hosmer–Leme show goodness-of-fit test, and overall predictive accuracy was assessed using the Brier score. To avoid overfitting, the multivariate model included clinically relevant variables and markers of disease severity.

Missing data were limited and primarily affected laboratory parameters at ICU admission. Simple imputation was performed prior to regression analyses to preserve cohort size, using median imputation for continuous variables and most-frequent category imputation for categorical variables. Given the retrospective design and low proportion of missingness, sensitivity analyses were not performed; however, the potential for residual bias related to missing data is acknowledged as a limitation.

Candidate variables for multivariate modeling included demographic factors, admission laboratory parameters, and clinically relevant ICU course variables. Collinearity between related variables (e.g., creatinine, AKI, hemodialysis) was assessed conceptually, and only variables providing complementary clinical information were retained. Continuous predictors were modeled linearly in the logit, acknowledging potential non-linearity as a limitation.

All statistical analyses were performed using IBM SPSS Statistics for Windows, version 30.0 (IBM Corp., Armonk, NY, USA). A two-sided *p*-value < 0.05 was considered statistically significant.

### 2.7. Ethical Considerations

The study was conducted in accordance with the Declaration of Helsinki and approved by the Ethics Committee of the Bihor County Emergency Clinical Hospital (Approval No. 36976/23.10.2023) and the Ethics Council of the same institution (Opinion No. 36746/20.10.2023). Due to the retrospective observational design, the requirement for informed consent was waived by the institutional ethics committees. All data were anonymized prior to analysis to ensure patient confidentiality.

## 3. Results

### 3.1. Study Population and Outcome in Patients with Diabetes Mellitus

A total of 443 critically ill adult patients with diabetes mellitus admitted to the intensive care unit during the study period were included in the analysis. All patients had a confirmed diagnosis of diabetes mellitus prior to or at the time of ICU admission, as defined by medical documentation, antidiabetic treatment, or diagnostic blood glucose–based diagnostic criteria.

The study population was stratified according to ICU outcome into survivors and non-survivors. Overall, 239 patients (54.0%) died during ICU hospitalization, while 204 patients (46.0%) survived until ICU discharge, indicating a high short-term mortality rate within the diabetic subgroup.

Patients with diabetes mellitus who did not survive ICU hospitalization were generally older compared to survivors. No major differences were observed in sex distribution between the two groups. Medical admissions predominated in both survivors and non-survivors, reflecting the clinical complexity and acute illness severity typical of diabetic ICU patients.

This stratification provided the basis for subsequent analyses focusing on laboratory parameters at ICU admission, the occurrence of acute complications, and the need for intensive care interventions in relation to short-term mortality among patients with diabetes mellitus.

Baseline characteristics of diabetic ICU patients stratified by survival status are presented in Table 1. Non-survivors were significantly older than survivors and were more frequently admitted for medical conditions. Sex distribution was similar between the two groups.

### 3.2. Laboratory Profile at ICU Admission in Diabetic Patients

At ICU admission, diabetic patients who did not survive exhibited a significantly altered laboratory profile compared to survivors. Non-survivors presented with higher markers of metabolic stress and impaired organ function, reflecting increased physiological vulnerability at the onset of critical illness.

Admission blood glucose and lactate levels were higher in non-survivors, while lymphocyte counts and renal function, reflected by higher serum creatinine levels were significantly lower compared to survivors. These differences suggest an association between early metabolic imbalance, immune dysregulation, renal dysfunction, and short-term mortality in critically ill patients with diabetes mellitus.

**Figure 1:**
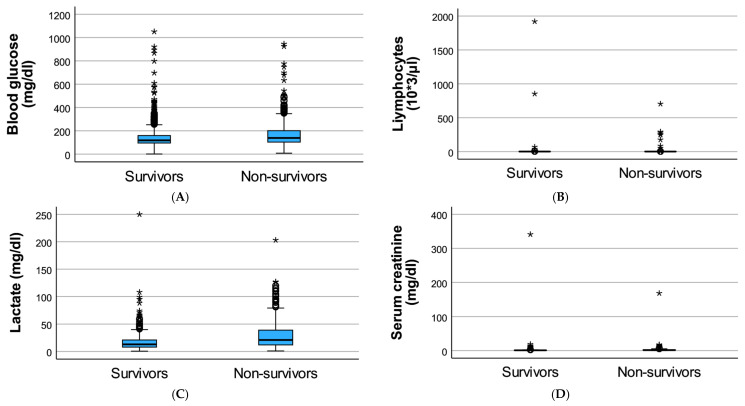
Laboratory parameters at ICU admission in diabetic patients according to survival status. Boxplots illustrate admission blood glucose (**A**), lactate (**B**), lymphocyte count (**C**), and serum creatinine (**D**) in critically ill patients with diabetes mellitus, stratified by ICU outcome (survivors vs. non-survivors). The central line represents the median, the box represents the interquartile range, and whiskers indicate data dispersion, * indicates outliers (values beyond 1.5 × IQR from the box).

The comparative laboratory profile of diabetic ICU patients according to survival status is summarized in Table 2 and Figure 1.

### 3.3. Acute Complications During ICU Stay in Patients with Diabetes Mellitus

The occurrence of acute complications during ICU hospitalization differed markedly between survivors and non-survivors among patients with diabetes mellitus (Table 3).

Non-survivors developed acute organ dysfunctions significantly more frequently than survivors. Acute respiratory failure was the most common complication overall and was observed in 76.2% of non-survivors compared to 46.1% of survivors. Similarly, mechanical ventilation was required in nearly all non-survivors (97.1%), while less than half of survivors required ventilatory support (39.2%).

Renal complications were also more prevalent among non-survivors. Acute kidney injury occurred in 38.5% of non-survivors compared to 22.1% of survivors, and the need for hemodialysis was almost four times higher in non-survivors (15.9% vs. 4.4%).

**Figure 2:**
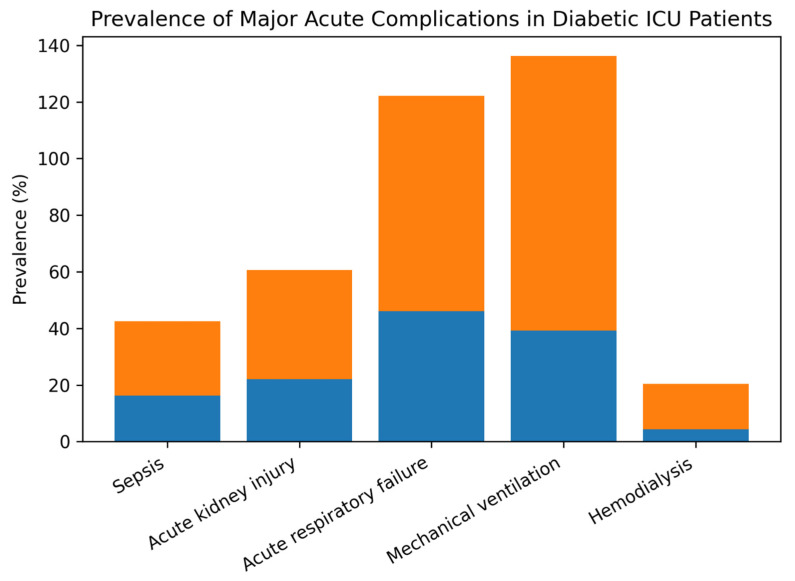
Prevalence of major acute complications in diabetic ICU patients according to survival status. The bar chart illustrates the prevalence (%) of major acute complications and intensive care interventions in critically ill patients with diabetes mellitus, stratified by ICU outcome (survivors vs. non-survivors). Blue bars represent survivors and orange bars represent non-survivors. Non-survivors exhibited a markedly higher prevalence of acute respiratory failure, acute kidney injury, sepsis, need for mechanical ventilation, and hemodialysis compared to survivors. Abbreviations: ICU, intensive care unit.

Infectious complications followed a similar pattern, with sepsis being significantly more frequent among non-survivors (26.4%) than survivors (16.2%). Overall, the presence of acute organ failure and the need for advanced ICU interventions were strongly associated with mortality in diabetic patients (Figure 2).

### 3.4. Predictors of ICU Mortality in Patients with Diabetes Mellitus

To identify predictors of ICU mortality among patients with diabetes mellitus, we performed univariate and multivariate logistic regression analyses. In univariate analyses, older age, higher lactate and serum creatinine levels, the presence of sepsis, acute kidney injury, acute respiratory failure, the need for mechanical ventilation, and hemodialysis were associated with increased odds of ICU death. Lower lymphocyte counts showed a borderline association with mortality.

In the multivariate model, lactate levels at ICU admission remained independently associated with mortality (OR = 1.02 per 1 mg/dL increase, 95% CI 1.00–1.04, *p* = 0.014). In addition, mechanical ventilation showed the strongest association with ICU mortality; however, this finding should be interpreted as reflecting advanced respiratory failure and overall disease severity rather than a direct causal effect (OR = 47.30, 95% CI 19.13–116.98, *p* < 0.001), and hemodialysis was also independently associated with mortality (OR = 3.38, 95% CI 1.13–10.11, *p* = 0.029). Other variables, including age, sepsis, acute kidney injury, acute respiratory failure, lymphocyte count, and serum creatinine, did not retain independent significance after adjustment, likely reflecting shared variance with markers of acute organ failure severity. Diabetes type was not consistently documented and was therefore not stratified.

The results of univariate and multivariate analyses are summarized in Table 4.

The multivariate model showed good discriminatory performance, with an AUC/ROC of 0.87. Model calibration was acceptable, as indicated by the Hosmer–Lemeshow test (χ^2^ = 12.63, *p* = 0.125). The overall probabilistic accuracy was supported by a Brier score of 0.135, suggesting good agreement between predicted and observed outcomes.

### 3.5. Model Performance and Risk Stratification

The performance of the multivariate logistic regression model was assessed in terms of discrimination and calibration. The model demonstrated good discriminatory ability, with an area under the receiver operating characteristic curve (AUC/ROC) of 0.87, indicating a high capacity to distinguish between survivors and non-survivors among diabetic ICU patients.

Model calibration was evaluated using the Hosmer–Lemeshow goodness-of-fit test, which showed no evidence of poor calibration (χ^2^ = 12.63, *p* = 0.125). Overall predictive accuracy was further supported by a Brier score of 0.135, reflecting good agreement between predicted probabilities and observed outcomes.

Taken together, these results highlight clinically relevant associations with ICU mortality; however, the model should be considered exploratory and requires external validation before use for formal risk stratification. The combination of early biological markers, particularly lactate levels, with indicators of advanced organ support allows for meaningful prediction of short-term ICU mortality in this high-risk population.

**Figure 3:**
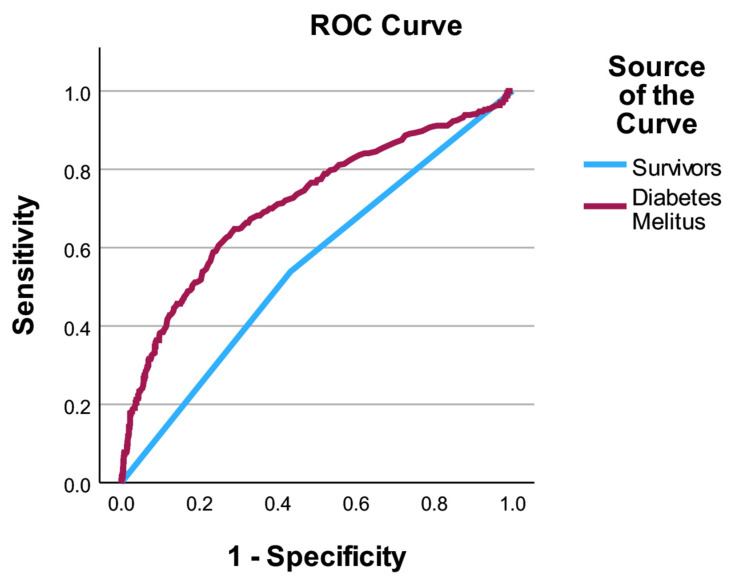
Receiver operating characteristic (ROC) curve for the multivariate model predicting ICU mortality in diabetic patients. The ROC curve illustrates the discriminatory performance of the multivariate logistic regression model in patients with diabetes mellitus, with an AUC of 0.87.

The discriminatory performance of the multivariate model is illustrated in Figure 3.

## 4. Discussion

In this study focusing exclusively on critically ill patients with diabetes mellitus, we identified a distinct clinical and laboratory profile associated with short-term ICU mortality. By restricting the analysis to diabetic patients, we were able to explore determinants of mortality beyond the mere presence of diabetes, highlighting factors that reflect metabolic stress, immune dysfunction, and acute organ failure.

At ICU admission, non-survivors exhibited significantly higher lactate levels, elevated blood glucose, impaired renal function, and lower lymphocyte counts compared to survivors. These findings suggest that diabetic patients who ultimately die during ICU hospitalization present with an early state of metabolic and immune vulnerability, which may predispose them to rapid clinical deterioration. Among these parameters, lactate emerged as an independent predictor of mortality, underscoring its role as a robust marker of tissue hypoxia, altered cellular metabolism, and global disease severity in critically ill patients with diabetes [14,15,16,17,18].

Acute organ dysfunctions occurring during ICU stay were markedly more frequent among non-survivors. Acute respiratory failure, acute kidney injury, and sepsis were all significantly associated with mortality in univariate analyses. However, in the multivariate model, mechanical ventilation and hemodialysis remained independently associated with ICU mortality. Importantly, these variables represent integrative markers of severe organ dysfunction and clinical deterioration during the ICU course, rather than modifiable prognostic factors on par with admission laboratory markers. Importantly, these interventions should not be interpreted as causal determinants of death but rather as integrative markers of disease severity and advanced organ failure. Their strong association with mortality reflects the profound physiological derangement present in patients requiring intensive organ support. This finding should be interpreted cautiously, as these interventions represent markers of disease severity rather than direct causal factors. Their strong association with mortality reflects the profound physiological derangement present in patients requiring advanced organ support [19,20,21,22]. Their strong association with mortality reflects the profound physiological derangement present in patients requiring advanced organ support.

The loss of statistical significance for several biological and clinical variables in the adjusted model likely reflects collinearity and shared variance with markers of severe organ failure. For example, renal dysfunction, sepsis, and respiratory failure frequently coexist in critically ill diabetic patients, forming a cascade of interrelated pathophysiological processes. In this context, mechanical ventilation and hemodialysis may act as integrative indicators of multisystem failure, capturing the cumulative burden of acute illness more effectively than individual variables [23,24,25,26,27].

Importantly, the good discriminatory performance of the multivariate model (AUC = 0.87) suggests that a combination of early biological markers and ICU interventions can provide meaningful prognostic information in diabetic patients. This supports the concept that risk stratification within the diabetic ICU population should account not only for baseline metabolic status but also for the rapid development of organ dysfunction requiring intensive support [28,29,30,31].

From a clinical perspective, these findings emphasize the importance of early recognition of metabolic stress and immune compromise in diabetic patients admitted to the ICU. Elevated lactate levels, lymphopenia, and early renal impairment may identify patients at particularly high risk, warranting closer monitoring and aggressive supportive management. Furthermore, the high mortality associated with advanced organ support highlights the need for preventive strategies aimed at mitigating progression to respiratory and renal failure in this vulnerable population.

### Limitations

Several limitations of this study should be acknowledged. First, the retrospective observational design precludes causal inference; therefore, the identified predictors should be interpreted as associations rather than direct determinants of ICU mortality. These variables may reflect the severity of critical illness rather than exert a causal effect on patient outcomes.

Second, this was a single-center study, which may limit the generalizability of the findings to other ICU settings with different patient populations, admission criteria, and clinical management protocols. Multicenter studies are warranted to validate these results across diverse healthcare systems.

Third, although key laboratory parameters at ICU admission were analyzed, data on chronic diabetes-related factors—such as HbA1c levels, duration of diabetes, and prior antidiabetic treatment—were not consistently available and could not be included. These factors may influence metabolic resilience and immune function during critical illness and should be considered in future prospective studies.

The use of single imputation rather than multiple imputation may have attenuated variance and influenced effect estimates; therefore, results should be interpreted cautiously.

Fourth, the study focused on short-term ICU mortality and did not assess post-discharge outcomes, long-term survival, or functional recovery. Consequently, the prognostic value of the identified predictors beyond the ICU stay remains uncertain.

Established ICU severity scores (e.g., SOFA, APACHE), detailed comorbidity indices, vasopressor use, and baseline renal function prior to admission were not consistently available and could not be incorporated into the multivariate model. As a result, residual confounding by illness severity cannot be excluded, particularly given the strong correlation between lactate levels, organ failure, and the need for intensive organ support.

Finally, some predictors included in the multivariate model, particularly mechanical ventilation and hemodialysis, should be interpreted as markers of advanced organ dysfunction and disease severity rather than direct causal factors. Their strong association with mortality reflects the extent of multisystem failure in critically ill patients with diabetes mellitus.

## 5. Conclusions

In this study of critically ill patients with diabetes mellitus, we identified a distinct set of biological and clinical factors associated with short-term ICU mortality. Diabetic patients who did not survive exhibited early signs of metabolic stress, immune dysregulation, and renal impairment at ICU admission, highlighting a state of increased vulnerability at the onset of critical illness.

Among the evaluated predictors, admission lactate levels and the need for advanced organ support, particularly mechanical ventilation and hemodialysis, emerged as independent determinants of ICU mortality. These findings underscore the importance of early biological assessment and close monitoring of diabetic patients at high risk of rapid clinical deterioration.

The model highlights clinically relevant markers associated with short-term ICU mortality; however, external validation and prospective evaluation are required before application for formal risk stratification. Future prospective and multicenter studies are needed to validate these findings and to explore targeted strategies aimed at mitigating metabolic and organ dysfunction in this vulnerable population.

## Figures and Tables

**Table 1:** Baseline characteristics of diabetic ICU patients according to survival status.

Variable	Total (*n* = 443)	Survivors (*n* = 204)	Non-Survivors (*n* = 239)	*p*-Value
Age, years (mean ± SD)	68.45 ± 12.94	65.85 ± 15.27	70.67 ± 10.07	<0.001
Median age, years	70	69	72	–
Male sex, *n* (%)	188 (42.4)	85 (41.7)	103 (43.1)	0.78
Female sex, *n* (%)	255 (57.6)	119 (58.3)	136 (56.9)	–
Medical admission, *n* (%)	327 (73.8)	122 (59.8)	205 (85.8)	<0.001
Surgical admission, *n* (%)	116 (26.2)	82 (40.2)	34 (14.2)	–

Abbreviations: ICU, intensive care unit.

**Table 2:** Laboratory parameters at ICU admission in diabetic patients according to survival status.

Parameter	Total (*n* = 443)	Survivors (*n* = 204)	Non-Survivors (*n* = 239)	*p*-Value
Blood glucose (mg/dL)	196.3 ± 84.7	182.4 ± 76.1	208.1 ± 89.3	<0.001
Lactate (mg/dL; 1 mmol/L ≈ 9 mg/dL)	28.6 ± 21.4	22.1 ± 17.8	34.1 ± 23.2	<0.001
Lymphocytes (10^3^/μL)	1.12 ± 0.61	1.28 ± 0.65	0.98 ± 0.54	<0.001
Serum creatinine (mg/dL)	1.87 ± 1.21	1.52 ± 0.94	2.18 ± 1.36	<0.001

Values are presented as mean ± standard deviation.

**Table 3:** Acute complications and ICU interventions in diabetic patients according to survival status.

Complication/Intervention	Total (*n* = 443)	Survivors (*n* = 204)	Non-Survivors (*n* = 239)
Sepsis, n (%)	96 (21.7)	33 (16.2)	63 (26.4)
Acute kidney injury, n (%)	137 (30.9)	45 (22.1)	92 (38.5)
Acute respiratory failure, n (%)	276 (62.3)	94 (46.1)	182 (76.2)
Mechanical ventilation, n (%)	312 (70.4)	80 (39.2)	232 (97.1)
Hemodialysis, n (%)	47 (10.6)	9 (4.4)	38 (15.9)

Abbreviations: ICU, intensive care unit.

**Table 4:** Predictors of ICU mortality in diabetic ICU patients (univariate and multivariate logistic regression).

Predictor	Unadjusted OR (95% CI)	*p*-Value	Adjusted OR (95% CI)	*p*-Value
Age (per 1-year increase)	1.03 (1.01–1.05)	<0.001	1.02 (0.99–1.04)	0.159
Male sex	1.06 (0.73–1.55)	0.762	0.98 (0.57–1.65)	0.926
Lactate (per 1 mg/dL increase)	1.03 (1.02–1.05)	<0.001	1.02 (1.00–1.04)	0.014
Lymphocytes (per 1 unit increase)	0.84 (0.70–1.00)	0.052	0.91 (0.80–1.04)	0.177
Serum creatinine (per 1 mg/dL increase)	1.20 (1.06–1.35)	0.003	1.08 (0.91–1.28)	0.364
Sepsis	1.85 (1.16–2.97)	0.010	1.04 (0.56–1.94)	0.898
Acute kidney injury	1.50 (1.01–2.23)	0.044	1.04 (0.63–1.72)	0.876
Acute respiratory failure	3.74 (2.49–5.60)	<0.001	1.60 (0.91–2.82)	0.103
Mechanical ventilation	60.19 (25.53–141.92)	<0.001	47.30 (19.13–116.98)	<0.001
Hemodialysis	4.10 (1.93–8.70)	<0.001	3.38 (1.13–10.11)	0.029

Skewed distributions observed. Abbreviations: OR, odds ratio; CI, confidence interval; ICU, intensive care unit.

## Data Availability

The raw data supporting the conclusions of this article will be made available by the authors on request.

## Abbreviations

AKI	acute kidney injury;
AUC	area under the curve;
CI	confidence interval;
DM	diabetes mellitus;
ICU	intensive care unit;
OR	odds ratio;
ROC	receiver operating characteristic;
SD	standard deviation
